# A Manual of Obstetrics

**Published:** 1887-03

**Authors:** 


**A Manual of Obstetrics.** By A. F. KING, A. M., M. D., Professor of Obstetrics and Diseases of Women in Columbia University, and in the University of Vermont, etc.,etc. With one hundred and two illustrations. Third edition. Philadelphia: Lea Bros. & Co. 1886.

Upon the appearance of the first and second editions of this work, we expressed a decided opinion as to its value. That a third edition is so soon called for, is evidence that the book has earned the approbation of both physicians and students. This last edition gives evidence of careful revision, and contains a number of new illustrations.

